# Gut Microbiota-Derived Short-Chain Fatty Acids in Inflammatory Bowel Disease: Mechanistic Insights into Gut Inflammation, Barrier Function, and Therapeutic Potential

**DOI:** 10.3390/ijms27021095

**Published:** 2026-01-22

**Authors:** Roberta Ottria, Susan Mirmajidi, Pierangela Ciuffreda

**Affiliations:** Dipartimento di Scienze Biomediche e Cliniche, Università degli Studi di Milano, 20157 Milan, Italypierangela.ciuffreda@unimi.it (P.C.)

**Keywords:** inflammatory bowel disease, gut microbiome, butyrate, short-chain fatty acids, intestinal barrier, inflammatory diseases

## Abstract

This review delves into the complex relationship between short-chain fatty acids (SCFAs) produced by the gut microbiota and inflammatory bowel disease (IBD). IBD, which includes Crohn’s disease and ulcerative colitis, is a group of chronic gastrointestinal disorders with an increasing global incidence. Despite extensive research, the exact etiopathogenesis remains elusive, although a complex interplay involving genetic predisposition, environmental influences, and abnormal immune responses against commensal gut microbes is widely recognized. SCFAs, primarily acetate and butyrate, emerge as key microbial metabolites derived from the fermentation of dietary fiber. They exert profound effects on gut homeostasis, notably with butyrate serving as an essential energy source for colonocytes, strengthening epithelial integrity, actively modulating local and systemic immune functions, suppressing the expression of pro-inflammatory cytokines, and enhancing mucosal defense mechanisms. However, clinical trials exploring SCFA administration have frequently yielded variable and inconsistent results due to differences in study design and patient characteristics. This review thoroughly analyzes the diverse roles of SCFAs in the large intestine, focusing on the intestinal barrier, immune modulation, and microbiota. It critically examines the therapeutic potential of SCFAs, including acetate and propionate, in addition to the well-known butyrate, in IBD management.

## 1. Introduction

Inflammatory bowel diseases (IBDs) represent a group of chronic disorders that impact the gastrointestinal system, which are primarily categorized into two main types: Crohn’s disease (CD) and ulcerative colitis (UC). The early 21st century has observed a significant increase in the incidence of IBD in newly industrialized nations. In the year 2019, the global count of IBD cases stood at 4.9 million. IBD is characterized by clinical manifestations including abdominal pain, diarrhea, and weight loss. Despite some recent therapies increasing quality of life for a portion of patients, the underlying causes and prevention of IBD continue to evade understanding [1].

Although the precise etiologies and underlying pathophysiological mechanisms of IBD are not fully understood, it is generally acknowledged that IBD results from a multifaceted interaction between genetic susceptibility and environmental factors. These interactions compromise the integrity of the mucosal barrier, disrupt the gastrointestinal microbiota, and precipitate dysregulated immune responses. Advances in genetics, immunology, and microbiology have significantly enhanced our understanding of the pathways involved in IBD like cytokine signaling (i.e IL-23, IL17, and TNF-α), innate immune responses (i.e., NF-κB), cellular stress, and epithelial barrier function [2,3]. The widely accepted theory is that IBD results from an abnormal and ongoing immune response to gut microbes or their byproducts, influenced by a host’s genetic predisposition and a weakened mucosal barrier [4]. The gut microbiome, a large community of microorganisms (including bacteria, viruses, and fungi) [5] in the human body, is critical for health, particularly in immune regulation, toxin removal, intestinal barrier integrity, and carbohydrate metabolism [6], as well as lipid absorption and energy storage [7,8,9]. Major projects like European Metagenomics of the Human Intestinal Tract (MetaHIT) and the Human Microbiome Project (HMP) have been instrumental in establishing baseline patterns of microbial diversity and distribution in the healthy gut [10]. In contrast, IBD often exhibits an altered microbial signature, characterized by a reduction in bacterial diversity, which is a hallmark of dysbiosis. Imbalances in the gut microbiota’s composition, known as dysbiosis, can greatly affect human physiology, making it a potential avenue for modulating and treating inflammatory conditions like IBD [3]. Moreover, gut bacteria ferment carbohydrates, often from dietary fiber, into short-chain fatty acids (SCFAs). The main SCFAs generated are acetate (approximately 60% of total SCFAs), propionate (about 20%), and butyrate (about 20%) [11]. These SCFAs, particularly butyrate, are important for gut health [12]. Butyrate, although often less plentiful than acetate, is a dynamic signaling molecule involved in balanced bacterial growth [13] and plays a key role in how mammals adapt gastrointestinal functions to their bacterial communities. Conventional therapeutic approaches primarily focus on symptom management through pharmacological interventions, including aminosalicylates, corticosteroids, immunomodulators, and biologics [14]. However, current IBD treatments often lead to recurrent inflammation and adverse effects, highlighting the urgent need for alternative or adjunctive therapeutic strategies. Given the crucial role of the gut microbiota in maintaining intestinal balance and its involvement in IBD, microbiome-targeted therapies, such as prebiotics, probiotics, antibiotics, and fecal microbiota transplantation (FMT), have been explored, though with inconsistent results [3]. This has driven interest in next-generation probiotics and microbiota-derived metabolites as potential therapeutic agents. Among these, SCFAs and SCFA-producing bacteria have shown promise in modulating gut microbiota and improving intestinal barrier function. Butyric acid supplementation has emerged as a potential strategy for mitigating IBD-related inflammation, offering a new avenue for therapeutic intervention.

## 2. Microbial Production, Influencing Factors, and Physiological Roles of SCFAs

**Figure 1:**
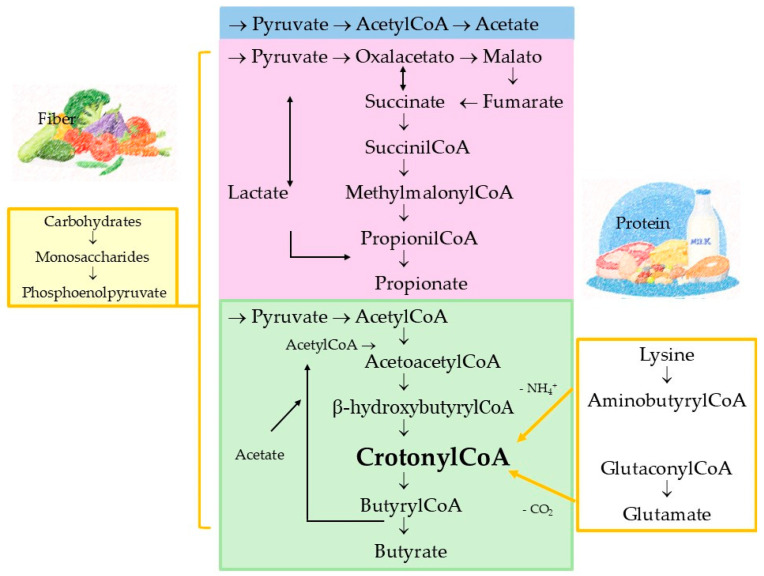
Metabolic pathways of dietary fiber and protein to short-chain fatty acids.

Several groups of gut bacteria are primarily responsible for the synthesis of SCFAs. Specifically, acetate is mainly produced by genera such as *Akkermansia*, *Bifidobacterium*, *Bacteroides*, *Prevotella*, *Ruminococcus*, *Clostridium*, *Streptococcus*, and the species *Blautia hydrogenotrophica.* Propionate largely originates from *Bacteroides*, *Dialister*, *Veillonella*, *Salmonella*, *Megasphaera elsdenii*, *Coprococcus catus*, *Roseburia inulinivorans*, *Phascolarctobacterium succinatutens*, and *Ruminococcus obeum* [15]. Finally, butyrate formation is mainly attributed to *Coprococcus eutactus*, *C. comes*, *Anaerostipes*, *Eubacterium rectale*, *C. catus*, *Eubacterium hallii*, *Roseburia*, and *Faecalibacterium prausnitzii* [16,17,18]. This production of SCFAs occurs through fermentation, primarily utilizing resistant starch and plant cell wall polysaccharides [19,20,21] as well as carbohydrates and proteins, within the favorable environment of the large intestine, to produce energy (Figure 1). As a result of this fermentation, the three main SCFAs generated via known pathways are acetate (C2, approximately 60% of total SCFAs), propionate (C3, about 20%), and butyrate (C4, about 20%) [11]. Moreover, it is important to highlight that acetate, propionate, and butyrate can also be synthesized from amino acids (Figure 1) [22], a pathway utilized by less than 1% of gut bacteria [23]. When carbohydrate availability is low, protein fermentation occurs, predominantly in the distal large intestine, producing not only SCFAs but also potentially harmful byproducts such as ammonia and sulfides [24]. Interestingly, butyrate can also be sourced from acetate conversion via glycolysis [25] and bovine milk fats. Additionally, undigested proteins and amino acids in the colon serve as supplementary substrates for acetate (i.e., glycine and ornithine) or butyrate (lysine) production by anaerobic bacteria [26]. Threonine and glutamate instead contribute to butyrate and acetate synthesis. Remarkably, threonine also acts as a key precursor in propionate formation [27], underlining the metabolic adaptability of gut bacteria in SCFA production.

Beyond dietary fiber, gut bacteria can also metabolize endogenous substrates such as exfoliated epithelial cells, mucus, and intestinal secretions that, in pathological conditions, may become available for microbial fermentation, potentially altering SCFA profiles. The amount and type of SCFAs produced in the gut are significantly influenced by a variety of factors. Their levels fluctuate throughout a person’s life, reflecting changes in the gut microbiome composition, which itself evolves with age and diet. In early life, *Bifidobacteria* dominate, leading to higher acetate production as they metabolize human milk oligosaccharides (HMOs) [28]. Later, after breastfeeding, there is an increase in *Firmicutes*, including families like *Lactobacillaceae*, *Ruminococcaceae*, and *Lachnospiraceae* [29,30], which can break down complex carbohydrates to produce butyrate and other SCFAs. In older age, the microbiota shifts again with a rise in *Enterobacteriaceae* [31]. Finally, pH level also significantly influences SCFA production by affecting both bacterial growth and the activity of enzymes involved [32].

**Figure 2:**
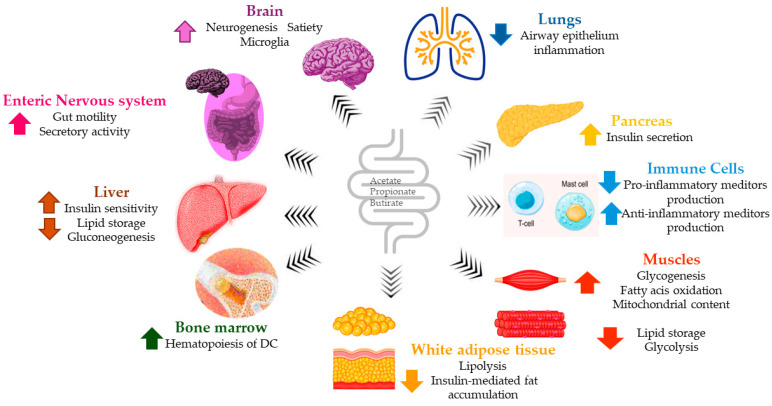
Systemic effects of SCFAs. These gut-derived metabolites regulate metabolism, immunity, and neural activity across organs, enhancing brain function, insulin secretion, and gut motility while reducing inflammation and improving metabolic balance.

SCFAs play distinct physiological roles [33]. Butyrate serves as an energy source for the gut mucosa, whereas propionate supports gluconeogenesis in the liver and interacts differently with host proteins and receptors [34]. The physiological effects of SCFAs are mainly mediated through Free Fatty Acid Receptors 2 (FFAR2) and 3 (FFAR3), G protein-coupled transmembrane receptors expressed in various cell types such as neurons, colonocytes, and adipocytes [35,36]. Specifically, acetate mainly activates FFAR2, while propionate primarily engages FFAR3, influencing diverse physiological processes such as inflammation modulation [34], neuronal energy metabolism [36], insulin secretion [37], and enteroendocrine signaling [38]. SCFAs are key regulators of the host immune response, influencing phagocytosis, chemokines, and central signaling pathways of cell growth and apoptosis, helping to shape the intestinal epithelial barrier. Moreover, SCFAs’ influence on host immune response potentially also impacts other organs via the gut–brain, gut–lung, and gut–liver axes. All these considerations suggest their possible pharmacological use in inflammatory diseases and infections [39] (Figure 2). SCFA dysregulation has also been associated with various neurological disorders—including Alzheimer’s disease, multiple sclerosis, Parkinson’s disease, depression, anxiety, autism spectrum disorder (ASD), and stroke—suggesting their potential involvement in neurophysiological homeostasis, influencing neurogenesis and microglia activation [40]. In particular, both propionic and butyric acid significantly influence gene expression related to neurotransmitters, cell adhesion, inflammation, oxidative stress, lipid metabolism, and mitochondrial function—all pathways implicated in ASD [41]. Interestingly, lipid and glucose homeostasis are also influenced by SCFAs through their involvement in insulin production and secretion. Propionate suppresses hepatic gluconeogenesis, while acetate and butyrate reduce lipogenesis and enhance leptin secretion. These beneficial metabolic effects, such as protection against diet-induced obesity and improved insulin sensitivity, are mediated through selective PPARγ modulation [42,43,44,45]. In fact, vinegar, which is rich in acetate, has been shown to reduce body weight, serum triglycerides, and fat mass [46]. Studies highlight, in vitro and in vivo, different roles of SCFAs in skeletal muscle, like their influence on energy metabolism and potential to increase skeletal muscle mass retention and to preserve oxidative phenotype. The metabolic link between SCFAs and skeletal muscles prompts the hypothesis of a gut–muscle axis [47]. Additionally, SCFA supplementation has demonstrated protective effects against liver steatosis in animal models [48]. Observational studies in humans also suggest a potential role of SCFAs in respiratory health, decreasing the risk of primary and secondary respiratory infections or modulating allergic airway exacerbations in animal models [49]. Finally, SCFAs reinforce intestinal barrier integrity with butyrate contributing to mucosal repair in colitis by stimulating “repairing” immune cells (M2 macrophages), which in turn enhance mucus production through the WNT-ERK1/2 signaling pathway [50]. Although in vitro and ex vivo studies have demonstrated that SCFAs influence oxidative stress, cellular differentiation, apoptosis, inflammation [51], and even cancer pathways via mechanisms such as histone acetylation and G protein receptor activation, strong clinical evidence supporting these effects remains limited [52]. A key metabolic function of SCFAs is their role in intestinal gluconeogenesis (IGN). Butyrate stimulates IGN via a cAMP-dependent mechanism, whereas propionate engages FFAR3 through a gut–brain circuit [53]. Moreover, metabolic benefits linked to SCFAs production or dietary fiber intake are lost in mice lacking IGN, highlighting their role in systemic glucose regulation.

Probiotics can modulate the gut microbiota and SCFA production, potentially offering benefits in various health conditions [11]. However, the effects appear to be strain-specific and context-dependent. Several in vitro and in vivo studies show that probiotic supplementation can alter the gut microbiota composition, often increasing beneficial bacteria like *Lactobacillus* and *Bifidobacterium*, and consequently influencing SCFA production. For example, a multi-strain probiotic increased lactate, leading to higher butyrate levels, while *Lactobacillus plantarum* P-8 increased acetate and propionate [54]. Moreover, fermented salami with *Lactobacillus rhamnosus* HN001 increased butyrate and reduced inflammatory markers in healthy individuals [55]. Additionally, colorectal cancer (CRC) is often associated with lower SCFA levels. Studies suggest that probiotics can inhibit cancer cell proliferation, potentially through increased SCFA production and other mechanisms like adhesion to cancer cells. In animal models, specific probiotics like *Butyrivibrio fibrisolvens* [56] or *Lactobacillus salivarius* reduced precancerous lesions and increased fecal SCFAs [57], suggesting that these probiotics can prevent CRC.

## 3. Role of Microbial SCFAs in Gastrointestinal Health: A Focus on IBD

**Figure 3:**
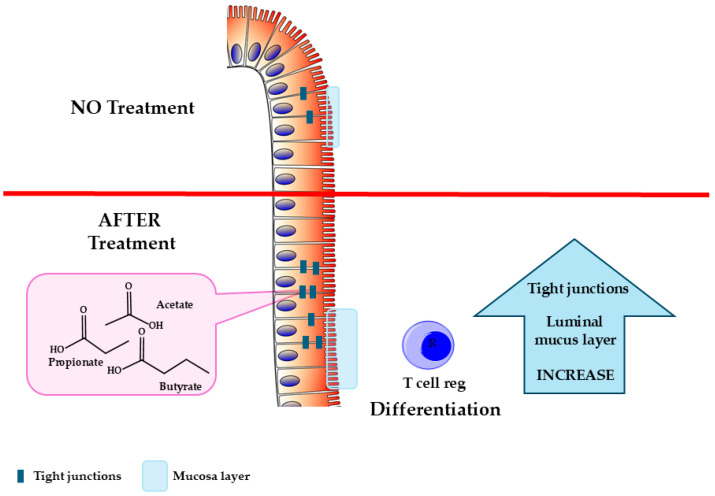
Effects of butyrate on intestinal barrier integrity.

The relationship between SCFAs and IBD is complex, involving interactions between gut microbiota, immune responses, and the integrity of the gut epithelial barrier [58,59]. IBD is classified into two subtypes: CD, which affects various parts of the digestive tract in a discontinuous manner, and UC, which is confined to the colon and can lead to severe complications such as ulcers, bleeding, and toxic megacolon. Studies indicate that individuals with IBD often show altered levels of SCFAs due to disruptions in gut microbiota composition and reduced fiber fermentation, leading to immune dysregulation and compromised gut barrier function [60]. SCFAs, particularly butyrate, have demonstrated potential benefits, exerting several important effects within the gut, in reducing inflammation and maintaining gut health; however, their precise role in IBD remains complex and requires further investigation [61,62,63]. In vitro studies suggest its promotion of epithelial proliferation in normal intestinal tissue [64] and its reduction in proliferation and induction of apoptosis in colonic cancer cells [65], potentially helping minimize colon cancer risk and progression. Additionally, SCFAs contribute to tissue repair by promoting epithelial cell proliferation and differentiation, aiding the healing of inflammation-induced damage [58]. Most importantly, butyrate strengthens the gastrointestinal epithelial barrier, which is especially relevant to IBD, a condition often associated with barrier dysfunction. SCFAs also help maintain the gut epithelial barrier by stimulating mucus production and reinforcing tight junctions between epithelial cells (Figure 3) [66], promoting regulatory T-cell (Treg) differentiation, and inhibiting Histone Deacetylases (HDACs) to support expression of genes involved in barrier and immune regulation.

Moreover, IBD is characterized by an abnormal immune response, where the immune system mistakenly attacks the gut lining, causing chronic inflammation. Beyond barrier function, butyrate exhibits strong anti-inflammatory properties by a variety of mechanisms, including Histone Deacetylase (HDAC) inhibition, immune cell activity modulation, and a reduction in the production of pro-inflammatory cytokines. Interactions with G protein-coupled receptors (GPCRs) such as GPR41, GPR43 and GPR109A, involved in immune function controlling [58,67], are also a mechanism of action of butyrate. In this context, SCFAs influence immune regulation by supporting regulatory T cells (Tregs), which suppress excessive immune reactions [68]. The pressing need for alternative or adjunctive therapeutic strategies to prevent and/or counteract the recurrence of inflammation in IBD has stimulated clinical research aimed at investigating SCFAs’ roles in intestinal physiology, with heterogeneous results. This review provides an in-depth analysis of the diverse roles of SCFAs in the human large intestine, with a focus on their involvement in intestinal barrier function, immune modulation, and microbiota composition. Although butyrate has received considerable attention for its anti-inflammatory and barrier-enhancing properties, emerging evidence highlights the complementary effects of other SCFAs, including acetate and propionate, in maintaining intestinal homeostasis. Additionally, this review integrates findings from clinical studies investigating the therapeutic potential of SCFAs in inflammatory IBD, providing a comprehensive perspective on their role in disease management. By elucidating these complex mechanisms, it aims to expand the field of microbiome-targeted interventions utilizing SCFAs, offering valuable insights into novel therapeutic strategies for IBD.

## 4. SCFAs in Intestinal Disorders: What Clinical Data Reveal

Starting from the last years of the 20th century, clinical research investigates the effects of SCFAs in IBD. As described before, administration of SCFAs or prebiotics, which can enhance SCFA production, has been effective in alleviating colitis and maintaining the mucosal barrier in UC patients. Additionally, a deficiency in SCFAs is likely to increase the risk of colitis [69], demonstrating, albeit in limited studies, that SCFAs play a beneficial role in disease management. We have compiled these results in Table 1.

The pioneering 1989 publication by Harig et al. [70] marked a significant turning point, as it demonstrated the successful application of SCFAs enemas in the treatment of diversion colitis. In the study, 5 patients were treated by administering SCFAs enemas containing sodium acetate (SA) (60 mM), sodium propionate (SP) (30 mM), and sodium n-butyrate (SB) (40 mM) twice daily for a period ranging from two to six weeks. As a control, the same patients also received isotonic saline enemas. After two weeks of SCFAs treatment, a visible endoscopic mucosal improvement was observed, with further improvement between four and six weeks. These results were mirrored by the histological analysis. Conversely, no beneficial effect was observed after a saline placebo. This pivotal study sparked considerable scientific curiosity, prompting additional research studies to validate Harig’s initial observations. Despite the promising initial results, subsequent clinical trials exploring SCFAs enemas, particularly in diversion and distal UC, have yielded highly variable and often inconsistent outcomes. Heterogeneity in study design, specific SCFAs formulations and dosages, patient characteristics, and a frequently observed notable placebo effect can be considered the main factors that influence this result variability. For better comprehension, the SCFAs clinical trials, together with the main results, are summarized below. Guillemot et al. [71] performed a randomized controlled trial involving 13 diversion colitis patients, with 7 receiving SCFAs enemas (SA 60 mM, SP 30 mM, and SB 40 mM twice daily) and 6 receiving isotonic saline as a placebo. The study concluded that after two weeks, SCFAs treatment did not result in significant endoscopic or histological improvement compared to the placebo. Additionally, from a bacteriological study in 1995 [76], the same authors found that SCFAs enemas did not induce significant changes in bacterial counts or species in comparison to placebo. However, the bacterial flora of diversion colitis patients differed from that of healthy controls (n = 16), suggesting that bacterial imbalance may be involved in the pathogenesis of the condition, A preliminary six-week study was conducted on 12 patients with distal colitis to test the usefulness of irrigation (100 mL of SA 80 mM, SP 30 mM, and SB 40 mM, twice daily) of the inflamed mucosa with SFACs [72]. Only 10 patients completed the trial, and 9 showed a statistically significant variation in mean disease activity index (DAI) score from 7.9 ± 0.3 to 1.8 ± 0.6 and in mucosal histology score from 7.7 ± 0.7 to 2.6 ± 0.7. Thus, the study concluded that patients with UC appear to benefit from increased contact with SCFAs critical energy substrates. In 1992, Senagore et al. [73] conducted a randomized study on 45 patients allocated to three treatment arms: corticosteroids (CS, n = 12), 5-aminosalicylic acid (5-ASA, n = 19), and SCFAs (n = 14). Baseline demographic data was similar across the groups. While all patients presented hematochezia and mucorrhea, which were resolved in six weeks in a large percentage of all groups, no significant differences were observed between the treatment arms for these symptoms or for endoscopic and histologic scores over time, although all groups improved. Recovery rates were also similar across the groups (CS: 10/12; 5-ASA: 17/19; SCFAs: 12/14), with a few instances of treatment failure or progression requiring alternative therapies. Notably, the cost analysis revealed significant cost savings with the SCFAs treatment compared to both CS and 5-ASA. Venia et al. [74] conducted a randomized, double-blind, placebo-controlled study in 40 patients (20 SCFAs, 20 placebo group) with mild-to-moderate distal colitis, investigating the efficacy of a six-week, twice daily, topical SCFA enema treatment (100 mL SA 80 mM, SP 30 mM, and SB 40 mM). The results only consider, for each group, patients characterized by an improvement associated with the treatment. A total of 14 SCFAs-treated patients, compared to 5 in the placebo group, showed significant improvements in all assessed parameters except bowel motions in the SCFA group. Statistically significant differences between the treatments were only noted for intestinal bleeding, urgency, and patient self-evaluation (possibly due to baseline imbalances). However, the study concluded that SCFAs enema irrigation is effective in distal colitis and may be a valuable alternative treatment. The effects of SCFAs on proliferation and endoscopic outcomes in colon proctomized patients with ileal pouches were also investigated [75]. A total of 16 UC patients (10 non-canalized pouches and 6 canalized pouches), as well as 9 familial adenomatous polyposis (FAP) patients (4 non-canalized pouches and 5 canalized pouches), were enrolled. Non-canalized patients received twice daily for one week, 30 mL of an SCFA solution (60 mM SA, 30 mM SP, 40 mM SB, and 22 mM sodium chloride), while patients with canalized pouches received the same treatment for two weeks. Mucosal biopsies were collected before and after treatment to assess proliferation. SCFAs treatment reduced proliferation only in UC patients with canalized pouches without significantly affecting inflammation or clinical functions in either group. Patz et al. [77] conducted a study on 10 refractory distal UC patients, treated twice daily with enemas (containing SA 60 mM, SB 30 mM, and SB 40 mM, pH7) for 6 weeks. The results showed a clinical response in 5 patients (50%), with a significant reduction in bleeding and endoscopic improvement, with 40% of patients rating the SCFAs treatment as superior to previous ones. The authors concluded that SCFAs enemas may be effective in refractory distal UC, highlighting the need for further controlled trials. In a subsequent randomized placebo-controlled clinical trial involving active UC patients, the potential benefits of SCFAs enemas, either a combination of three (SA 80 mM, SP 30 mM, and SB 40 mM) or SB monotherapy (100 mM), were evaluated, alongside maintenance oral anti-inflammatory medication [78]. The study involved 47 patients randomly assigned to one of three groups: SCFAs (n = 16), SB (n = 15), or placebo (n = 16). The volume of all three types of enemas was 60 mL, and the pH value was set at 5.5. The primary outcome, a reduction in the DAI by 3, did not show significant differences between the treatment groups after 3 weeks. Nevertheless, when considering complete response (final DAI < 3), a trend favoring SCFAs (47% remission) and butyrate (48%) over the placebo (25%) was noted. Only butyrate also significantly reduced the extent of affected colon at week 8. The study concluded that the sample size limited the ability to demonstrate a statistically significant advantage of SCFAs or butyrate on the primary endpoint, likely due to the notable placebo effect observed. Moreover, a possible benefit of SCFAs or butyrate to secondary endpoints is suggested, justifying larger, adequately powered studies, estimated at 70 patients per group. Breuer and coworkers (Breuer, Soergel et al. 1997), in 1997, Ref. [79], proposed a double-blind placebo-controlled trial on distal UC patients to examine the effects of topical SCFAs applied directly to the 103 patients were enrolled and treated twice a day for 6 weeks. Treatment consisted of 100 mL SCFAs solution (SA 80 mM, AP 30 mM, and SB 40 mM, pH 7) or placebo (sodium chloride 140 mM, pH 7). Out of the 91 people who finished the six weeks of treatment, more people in the SCFAs group showed clinical and histological, non-statistically significant improvements (33% vs. 20%). The results could have been affected by patients’ adherence to the treatment; indeed, improvements in the SCFAs group were associated with at least five out of the six irrigations prescribed, while only 37% of those who did not improve were as compliant. Interestingly, the 42 short current UC patients (less than 6 months) seemed to show higher sensitivity to SCFAs, showing a better response (48% improving compared to only 18% on placebo) and larger decrease in their clinical activity scores. Moreover, an open label extension of the trial allowed the placebo group to obtain SCFAs for an additional 6 weeks, with patients reporting clinical improvement and DAI reduction (65%). However, the data suggests efficacy only in a subset of patients. To our knowledge, the latest work in the literature using SCFAs is the double-blind crossover study of Schauber in 2000 [80]. The study investigated the effect of SCFAs enemas on inflammation in the excluded rectums of patients previously treated with colectomy for acute colitis in 9 patients with Hartmann-closed rectums (8 CD, 1 UC). Either SCFAs (60 mL of SA 80 mM, SP 30 mM, and SB 40 mM, pH 7.0) or a placebo (60 mL of isotonic NaCl) were administered twice daily for 3 weeks. Both solutions contained methylparahydroxybenzoate as a preservative. Results showed no beneficial effect on inflammation. However, an altered rectal flora was observed in the SCFAs group compared to the controls.

In conclusion, while some clinical improvements were observed, the results varied considerably due to differences in study design, patient characteristics (particularly refractoriness to previous treatments), SCFAs dosages and formulations, administration protocols, and the size of the placebo effect, highlighting the need for further well-designed research to optimize SCFAs therapy.

## 5. The Therapeutic Potential of Butyrate in Inflammatory Bowel Disease (IBD)

Numerous studies have demonstrated the potential effects of oral butyrate supplementation in reducing gut inflammation. Butyrate plays a key role in the remission of IBD and is associated with maintaining intestinal integrity and reducing inflammatory biomarkers. Many studies have been published about the potential effects of various butyrate treatments, like oral supplements and enemas, demonstrating their effectiveness in the treatment of IBD.

### 5.1. Oral Butyrate Administration and Efficacy

While certain studies using oral microencapsulated sodium butyrate (BLM) have shown limited or no significant effects on clinical activity in active UC, a more promising clinical application lies in the successful maintenance of remission, where significant long-term benefits have been observed in UC patients.

Among the various forms of butyrate administration, oral BLM supplementation appears to be a valid therapy for maintaining remission in patients with UC. Facchin et al. [81], in a double-blind, placebo-controlled study involving 49 IBD patients (19 CD and 30 UC), reported that adding oral BLM supplementation (1800 mg/d) to standard therapy in IBD patients altered the gut microbiota. This alteration involved an increase in SCFAs-producing bacteria (such as *Lachnospiraceae* spp. in UC) or butyrogenic colonic bacteria (*Butyricicoccus* in CD), suggesting a potentially anti-inflammatory mechanism. No effects on clinical activity between the two groups (assessed by Mayo score, Harvey–Bradshaw Index, IBD questionnaire (IBDQ)) were observed. Another study [82], focusing on 39 UC patients in clinical remission, found significant long-term benefits. A total of 83.3% of patients treated BLM (1 g/d for 12 months) as an add-on therapy to standard melamine showed remission maintenance (Mayo partial score ≤ 2 and fecal calprotectin (FC) < 250 µg/g) compared to 47.6% in the control group.

Another promising strategy for SB delivery is the development of coated tablets, ^13^C-SB with HPMC and Shellac, which were designed to ensure targeted and delayed release in the ileo-cecal or colon region and assessed. SB-targeted release was confirmed through breath testing (^13^CO_2_) and tauroursodeoxycholic acid (TUDCA), co-administered to assess absorption. Butyrate intestinal release by coated tablets demonstrated a delayed release of ^13^C-butyrate (^13^CO_2_ in breath test) by 2–3 h with respect to the uncoated tablet. Absorption was evaluated by the enzymatic quantification of TUDCA in plasma samples for 8 h. The study considers both healthy individuals and CD patients, including those with variable intestinal transit times [83]. The pilot study by Vernia et al. [84] was a six-week randomized, double-blind, placebo-controlled trial that evaluated the safety and efficacy of oral butyrate-coated tablets combined with standard oral mesalazine (2.4 g/day) in 30 patients with mild-to-moderate UC. The butyrate dosage used was relatively high, 4 g/day, representing 35–60% of the normal daily intracolonic butyrate production. A key finding was the formulation’s safety and excellent tolerance, with no untoward side effects reported other than occasional foul-smelling belching in two patients. Although all clinical, endoscopic, and histologic scores were significantly improved in both the combined (butyrate + mesalazine) and mesalazine-alone groups, the difference in efficacy between the two groups was not statistically significant over the short six-week period. Crucially, however, the combined therapy group showed a significantly better improvement versus baseline values when assessing both the clinical index and the UC disease activity index (UCDAI). These data suggested that oral butyrate may improve the efficacy of oral mesalazine in active UC. In a pilot study of 13 patients with mild-to-moderate ileo-colonic CD, oral butyrate-coated tablets (4 g/day for 8 weeks) were demonstrated to be safe and well-tolerated. Treatment induced clinical improvement in 69% and remission in 53% of patients (lasting 2 weeks post-treatment), significantly reduced leukocyte count and erythrocyte sedimentation rate (ESR), improved endoscopic and histologic scores at the ileo-caecal level, and decreased mucosal NF-κB and IL-1β expression, suggesting anti-inflammatory effects via NF-κB downregulation [85].

Only one study involving a pediatric population is reported. The study analyzed 72 patients (aged 6–18 years) with colonic CD or UC, who received standard therapy and were randomized to receive 300 mg/day of SB or placebo for 12 weeks. Many patients achieved remission in 12 weeks, although no differences in remission rate or median disease activity were observed between groups. Disease activity was assessed using the Pediatric CD Activity Index (PCDAI) for CD patients and the Pediatric UC Activity Index (PUCAI) for UC patients [86].

Finally, Firoozi and coworkers [87] conducted a randomized placebo-controlled trial evaluating the effects of a 12-week treatment with 600 mg per day of oral SB in 36 patients with active mild-to-moderate UC. Results showed significant benefits in three main areas: a marked reduction in key inflammatory biomarkers (fecal calprotectin and high-sensitivity C-reactive protein), an upregulation of circadian clock genes (including CRY1, CRY2, PER1, and BMAL1), and a notable improvement in sleep quality and overall quality of life. This highlights oral SB as a safe and effective adjunct therapy that modulates circadian gene expression and reduces inflammation to enhance patient well-being.

### 5.2. Topical Administration (Enemas)

Topical administration of butyrate has also been investigated, particularly for refractory distal UC, leading to inconsistent results (Table 2).

#### 5.2.1. Efficacy in Refractory UC

A randomized, single-blind, placebo (100 mL NaCl 140 mM)-controlled trial investigated topical SB (100 mL, SB 100 mM, and NaCl 40 mM) administered twice a day in 10 patients with refractory distal UC. Butyrate treatment led to significant clinical remission, evidenced by a decrease in stool frequency (from 4.7 to 2.1 per day), cessation of fecal bleeding (9 patients), and improvement in endoscopic and histological inflammation scores [88]. Moreover, treatment normalized the pathological expansion of the upper crypt proliferative zone, supporting the view that butyrate deficiency plays a role in UC pathogenesis and that restoring local metabolic supply is therapeutic. Despite these positive findings, a placebo-controlled trial investigating the efficacy of nightly 60 mL SB enemas (80 mM) in 38 patients with active distal UC revealed butyrate inefficacy. Based on the hypothesis that UC is related to an energy-deficient state linked to reduced SCFA production or utilization, the authors concluded that the butyrate treatment was not efficacious. Indeed, the data highlighted statistically similar clinical improvement (37%) and remission rates (16%) compared to the placebo group (47% improvement, 16% remission) [89]. Differently, Vernia and coworkers demonstrated the efficacy of SB as an adjuvant to aminosalicilates (5-ASA) in refractory UC. Specifically, for refractory distal UC, topical combination therapy utilizing SB and 5-ASA has demonstrated robust efficacy, achieving significantly better results and higher remission rates than 5-ASA alone or SB monotherapy. At first, authors demonstrated that intra-rectal instillation of combined 5-ASA and SB (100 mL SB 80 mM containing 2 g 5-ASA) was shown to be a useful therapeutic tool for refractory distal UC, with marked clinical and endoscopical improvement observed in seven of nine unresponsive patients [90]. Crucially, the clinical improvement occurred earlier (usually within the second week) than previously reported butyrate monotherapy, suggesting the combination increases the effectiveness of the individual treatments. Some years later, the same authors presented a six-week double-blind, placebo-controlled multicenter trial investigating the efficacy of topical SB in 51 patients with distal UC who were unresponsive to standard topical 5-ASA or steroid therapy. Participants were randomly assigned to receive either a combination of topical 5-ASA with SB (80 mL SB 80 mM containing 4 g mesalazine) or 5-ASA with saline (80 mL saline containing 4 g mesalazine), administered twice daily. The SB group showed significantly better results, higher remission rates, improved clinical symptoms (such as reduced bowel movements and urgency), and more positive self-assessments. The findings suggest the therapeutic value, without adverse effects, of topical butyrate in managing refractory UC [91].

#### 5.2.2. Mechanistic Insights from Enemas

The study of Lührs et al. [92] explored the effects of twice daily, 60 mL SB enemas (100 mM, pH 5.5, iso-osmotic, retained ≥30 min) versus isotonic-saline placebo over 8 weeks in 11 patients with active distal UC (DAI > 3) who maintained stable oral mesalazine, sulfasalazine, or ≤25 mg/day corticosteroids and had discontinued all rectal therapies for at least 4 weeks. The treatment led to a marked reduction in lamina propria macrophages displaying nuclear translocation of NF-kB, a change sustained at both 4 and 8 weeks and closely linked to clinical and histological improvements. Histologically, butyrate also reduced neutrophil infiltration in the crypt and surface epithelia, as well as the number of lamina propria lymphocytes and plasma cells. Patients receiving butyrate also showed a significant decrease in DAI compared to baseline and to the placebo group after eight weeks, supporting its anti-inflammatory potential.

Otherwise, daily rectal butyrate (60 mL 100 mM SB or saline) enemas administered for 20 days to 35 UC patients in remission exerted only minor effects on colonic inflammation (e.g., increasing the IL-10/IL-12 ratio and CCL5 colonic concentration) and showed no significant overall effects on oxidative stress parameters [93]. The same authors also aimed to evaluate the in vivo effect of SB enemas on the human colonic mucus layer, analyzing mucin glycoprotein (MUC2) trefoil factor (TFF3) and secretory IgA in biopsies or fecal samples. A total of 35 UC patients in clinical remission and 16 healthy volunteers were enrolled to self-administer SB (60 mL 100 mM or placebo) once daily for 2–3 weeks. Results showed that the intervention did not significantly modulate the expression of MUC2 or TFF3 in either healthy or UC patients, nor did it affect the percentage of total mucin secretion or secretory IgA concentrations in either groups [94]. Finally, a randomized, double-blind, placebo-controlled study investigated topical administration of high concentrations of SB enemas (2 g/30 mL; 600 mM). A total of 20 patients operated on at least 30 days earlier for diverticular disease, cancer, or IBD were enrolled and randomly allocated to SB or a placebo (0.01 g/30 mL 3 mM) and enemas, treated twice daily for 30 days. SB significantly improved endoscopic scores and reduced mucosal atrophy in patients with diverted colorectal mucosa, suggesting the treatment prevents atrophy and improves tissue integrity recovery via the up-regulation of mucosal repair genes, including bone morphogenetic protein (BMP) antagonists [95].

## 6. Future Directions and Perspectives

The intricate relationship between dietary factors, the gut microbiota, and SCFAs in IBD presents a promising yet complex area for future research and therapeutic development. While current evidence highlights the crucial role of SCFAs in maintaining intestinal balance and modulating inflammation, inconsistencies in clinical trial outcomes underscore the need for more rigorous and targeted investigations.

### 6.1. Optimizing SCFA-Based Interventions and Dietary Strategies

Current research on SCFAs in colitis has yielded varied results due to differences in study design, patient characteristics, specific SCFA dosages and formulations, and administration protocols, emphasizing the need for further well-designed studies to optimize SCFA therapies. Beyond direct SCFA administration, future studies should focus on identifying which specific components or types of dietary fiber, such as resistant starch or wheat bran, confer the most benefit to moving to general dietary recommendations associated with supplementation. For instance, despite findings that increasing resistant starch and wheat bran intake in UC patients tended to normalize gut transit and improve delivery of fermentation products, it did not correct fermentative deficiency.

### 6.2. Elucidating Mechanisms and Understanding Heterogeneity

A critical direction for future research is to clarify the exact mechanisms through which SCFAs and SCFA-producing dietary interventions exert their anti-inflammatory and barrier-enhancing effects. This includes understanding the complex interplay between nutritional, biliary, and microbial dynamics in diet-related diseases. Furthermore, the observation that the efficacy of dietary interventions can differ between CD and UC, potentially due to variations in SCFA generation and saccharolytic potential of their respective microbiomes, necessitates further studies. Investigating the impact of medications, such as mesalazine, on carbohydrate fermentation by fecal microbiota also warrants examination.

### 6.3. Advancing Personalized Nutrition and Biomarker Discovery

The heterogeneity of patient responses to dietary interventions, increased SCFA production, or SCFA direct administration highlights the importance of personalized approaches. Future studies should aim to identify factors that predict success in these interventions, including disease type, disease duration, and prior exposure to biologics. This involves evaluating mucosal healing and examining changes in the microbiome with dietary therapy to identify which patients are most likely to benefit.

### 6.4. Improving Study Designs and Long-Term Assessments

To yield more reliable results and inform clinical recommendations, future research requires larger sample sizes, longer follow-ups, and robust comparative effectiveness designs. This includes conducting well-designed randomized controlled trials for dietary interventions or supplementation, alone or in addition to standard therapy, ideally comparing them with existing medical therapies. Furthermore, addressing the question of endurance for dietary interventions is vital; long-term studies, including assessments after the termination of dietary changes, are necessary to determine if beneficial effects are sustained.

To address all these main points, a multi-omics approach combining metagenomics, metabolomics, and transcriptomics could be a powerful tool to investigate the simultaneous effects of SCFAs on microbiota modulation, SCFA production, and important inflammatory (i.e., Calprotecine, IL-23, IL17, and TNF-α) and intestinal permeability biomarkers (i.e., Zonulin, Occludin, or Claudines).

## 7. Conclusions

This comprehensive review has highlighted the crucial role of SCFAs—particularly butyrate, propionate, and acetate—as potent metabolites derived from the gut microbiota that are essential for maintaining intestinal homeostasis. Butyrate exerts profound effects by serving as a primary energy source for colonocytes, strengthening epithelial integrity, and actively modulating local and systemic immune functions. However, the efficacy of SCFA-based interventions in clinical practice remains ambiguous. As detailed by our analysis, despite promising initial results, subsequent clinical trials utilizing SCFA enemas in distal UC and diversion colitis have shown considerable variability and inconsistency due to differences in study design, patient characteristics, specific dosages and formulations, and frequently observed, notable placebo effects in these trials. Furthermore, while certain studies using oral BLM have shown limited or no significant effects on clinical activity in active UC, a more promising clinical application lies in the successful maintenance of remission, where significant long-term benefits have been observed in UC patients. Specifically for refractory distal UC, topical combination therapy utilizing sodium butyrate alongside 5-ASA has demonstrated robust efficacy, achieving significantly better results and higher remission rates than 5-ASA alone.

In conclusion, advancing the field of microbiome-targeted interventions using SCFAs and/or dietary approaches in IBD requires comprehensive, interdisciplinary research that addresses current limitations. Clarifying the precise mechanisms through which SCFAs exert anti-inflammatory and barrier-enhancing effects employing robust methodologies such as larger, well-designed randomized controlled trials with mucosal healing incorporated as a definitive endpoint is of fundamental importance for future studies. The development of personalized strategies that account for patient heterogeneity—including disease type and duration—is also required. By integrating these elements, future studies hold the potential to translate promising preliminary findings into effective, evidence-based dietary guidelines and innovative therapeutic strategies for IBD management.

## Figures and Tables

**Table 1:** Key features of SCFAs administration studies of IBD.

SCFA Treatment Parameters	Colitis Model	Effects	Health Outcomes	Author(s)
60 mL of SCFAs (60 mM SA, 30 mM SP, 40 mM SB) via instillation, twice daily, 2–6 weeks.	4 diversion colitis, specifically in segments of the colorectum after surgical diversion of the fecal stream.	SCFAs resolved symptoms and inflammation, endoscopic mucosal improvement after 2 weeks and improvement at 6 weeks.	Remission of diversion colitis. Histological improvements.	Harig 1989 [70]
60 mL rectal enemas containing SA (60 mM), SP (30 mM), and SB (40 mM) twice daily for 14 days.	13 diversion colitis (post-surgical fecal stream diversion)	No significant endoscopic or histologic improvement after 2 weeks compared to saline placebo.	No difference between SCFAs and placebo groups after 2 weeks of treatment.	Guillemot 1991 [71]
Rectal irrigations with 100 mL of SCFAs SA (80 mM), SP (30 mM), and SB (40 mM), twice daily, 6 weeks.	12 distal UC	SCFAs led to significant clinical and histological improvement in 9 out of 10 patients; decrease in disease activity index and mucosal histology score.	Clinical improvement: reduced stool frequency, rectal bleeding, and improvements in mucosal appearance and impact on daily activities. Histological improvements.	Breuer 1991 [72]
60 mL of SCFA solution (SA 60 mM, SP 30 mM, and SB 40 mM) per rectum twice daily, 6 weeks.	14 idiopathic proctosigmoiditis	SCFAs enemas were equally efficacious to corticosteroid (CS) or 5-ASA enemas for the treatment of proctosigmoiditis.	Recovery occurred in a similar proportion in each of the three groups; SCFAs had significantly lower treatment cost.	Senagore 1992 [73]
100 mL twice daily enemas of SCFAs (SA 80 mM, SP 30 mM, and SB 40 mM) for 6 weeks.	40 mild-to-moderate distal UC	SCFAs enemas were effective in improving clinical, endoscopic, and histological findings.	Improvement in several clinical parameters: intestinal bleeding, urgency, and patient self-evaluation score.	Vernia 1995 [74]
30 mL of an SCFAs solution (60 mM SA, 30 mM SP, 40 mM SB, and 22 mM sodium chloride, pH 7) twice daily for 1 or 2 weeks. 4 familial adenomatous polyposis (FAP) patients with noncanalized pouches, 5 FAP patients with canalized pouches. Treated with the same solution and duration as the UC.	10 UC noncanalized pouches and 6 UC canalized pouches	SCFAs reduced proliferation only in UC canalized pouches. SCFAs did not alter proliferation in FAP in either type of pouch.	SCFAs did not control inflammation and clinical functions, but reduced cell proliferation in UC patients with canalized pouches. FAP patients were refractory to SCFAs treatment.	Tonelli 1995 [75]
60 mL rectal enemas with SCFAs SA (60 mM), SP (30 mM), and SB (40 mM) twice daily for 14 days.	13 diversion UC following surgical fecal stream exclusion	No significant changes in bacterial species or total counts.	SCFAs enemas did not improve microbial flora; findings support a role of dysbiosis in diversion colitis pathogenesis rather than SCFA deficiency alone.	Neut 1995 [76]
Enemas containing SA 60 mM, SP 30 mM, and SB 40 mM (titrated to a pH of 7) twice daily for 6 weeks.	10 refractory distal UC	Clinical response in 50% of patients; significant reduction in bleeding and tenesmus; endoscopic improvement in 5 patients.	Clinical remission in 4 patients, with decreased bleeding and tenesmus. Endoscopic improvement in 5 patients. No histological improvement.	Patz 1996 [77]
Rectal enemas of SCFAs SA 60 mM, SP 30 mM, and SB 40 mM, or SB alone (100 mM), or a saline placebo (isotonic saline) twice daily for 8 weeks.	47 distal UC	DAI decreased significantly in all treatments with no difference among groups. No differences in endoscopic mucosa and histologic degree of inflammation. At 8 weeks, fewer colonic segments were affected endoscopically by butyrate than placebo treatment.	Possible benefit in remission rates and colon involvement; study limited by small sample size and notable placebo effect.	Scheppach 1996 [78]
Rectal enemas contained SA (80 mM), SP (30 mM), and SB (40 mM) adjusted to pH 7 twice daily for 6 weeks.	103 distal UC	No statistically significant difference in overall improvement vs. placebo; greater symptoms and histologic score reductions in SCFAs group (not significant); significant benefit in patients with flare-ups < 6 months; adherence linked to improvement.	SCFAs enemas were not of therapeutic value in this controlled trial, but results suggest efficacy in subsets of patients with distal UC with short active episodes.	Breuer 1997 [79]
Rectal enemas containing SA (80 mM), SP (30 mM), and SB (40 mM) twice daily for 4 weeks.	103 Hartmann-closed rectum after colectomy for acute UC	No significant differences in symptoms, mucosal inflammation, histology, or microbiology between SCFAs and placebo.	SCFAs enemas had no beneficial effect on inflammation in excluded rectum in patients after colectomy for colitis.	Schauber 2000 [80]

5-Aminosalicylic acid (5-ASA), sodium acetate (SA), sodium propionate (SP), sodium butyrate (SB), short-chain fatty acids (SCFAs), ulcerative colitis (UC).

**Table 2:** Sodium butyrate: therapeutic potential in IBD (CLINICAL TRIALS).

Treatment	Desease	Effects	Health Outcomes	Reference
Oral BLM 3 cps/d (1800 mg/d), or placebo, 2 months in addition to standard therapy	30 UC and 19 CD	SB altered gut microbiota by increasing bacteria able to produce SCFAs in UC patients (*Lachnospiraceae* spp.) and butyrogenic colonic bacteria in CD patients (*Butyricicoccus*), no statistical significance in FC.	No effects on clinical (Mayo score, Harvey–Bradshaw Index, IBDQ).	Facchin 2020 [81]
Oral cps (500 mg) BLM 2 cps/day plus standard mesalamine, control group standard mesalamine, 12 months	39 UC	Mayo partial score ≤ 2, FC < 250 µg/g at 12 months (64.1%patients, 83.3% in BLM group −47.6% in control).	72.2% of BLM improved residual symptoms compared to 23.8% of controls. Clinical remission maintenance.	Vernero 2020 [82]
Oral 1 g SB coated tablets. 500 mg tauroursodeoxycholic acid (TUDCA) was co-administered as a biomarker	12 healthy subjects and 12 CD	The coated formulation delayed the release of ^14^C-butyrate by 2–3 h compared to uncoated tablets. SB was released uniformly in the ileocecal region and colon.	Oral co-administration of SB-mesalazine appears to improve the efficacy of mesalazine in active UC.	Roda 2007 [83]
Group A: oral SB, 4 g/day +mesalazine 2.4 g/day. SB tablets of 800 mg. Group B: oral mesalazine at 2.4 g/day + placebo. 6 weeks	30 mild-to-moderate active UC	All clinical, endoscopic, and histologic scores significantly improved in both treatment arms compared to baseline. Group A significantly better improvement vs. baseline for clinical index and UCDAI score.	7 patients in Group A achieved remission (UCDAI < 3). Group A showed a significantly better improvement vs. baseline values (*p* < 0.05) for the clinical index and the UCDAI score.	Vernia 2000 [84]
Oral coated tablets 4 g SB/day, 8 weeks	13 CD	Leucocyte blood count, erythrocyte sedimentation rate and NF-kB and IL-1b mucosal levels significantly decreased in 9 patients.	3 patients showed no clinical improvement. 9 patients (69%) responded to treatment, 7 (53%) achieved remission, 2 partial responses. Endoscopic and histological score significantly improved after treatment.	Di Sabatino 2005 [85]
Oral SB (150 mg twice a day, total 300 mg/day), administered for 12 weeks	72 pediatric CD or UC (aged 6–18 years) SB: 29 Placebo: 43	No statistically significant difference regarding remission rate (*p* = 0.371), median disease activity (PCDAI/PUCAI scores), or median fecal calprotectin concentration (*p* = 0.466). No patients reported adverse events.	A 12-week supplementation with SB, as adjunctive therapy, did not show efficacy in newly diagnosed children and adolescents.	Pietrzak 2022 [86]
12 weeks; oral SB (600 mg/day) vs. placebo	36 UC. SB: 18 Placebo: 18	Significantly in FC and high-sensitivity C-reactive protein compared to the placebo (*p* < 0.001).	Significant and more substantial decrease in the Pittsburgh Sleep Quality Index (PSQI) score (*p* < 0.001). A significant improvement in the overall Inflammatory Bowel Disease Questionnaire-9 (IBDQ-9). No significant adverse effects.	Firoozi 2024 [87]
2-week course of SB enemas (100 mM), or placebo (140 mM NaCl), administered in a single-blind, randomized, crossover trial.	10 distal UC unresponsive or intolerant to standard therapy for at least 8 weeks	SB significant reductions in inflammation: the endoscopic score from 6.5 ± 0.4 to 3.8 ± 0.8 (*p* < 0.01), histological degree of inflammation from 2.4 ± 0.3 to 1.5 ± 0.3 (*p* < 0.02). SB significantly normalized pathological proliferation pattern by reducing the upper crypt-labeling index from 0.086 ± 0.019 to 0.032 ± 0.003 (*p* < 0.03).	Clinical symptoms improved significantly: stool frequency decreased from 4.7 ± 0.5 to 2.1 ± 0.4 defecations per day (*p* < 0.01). Discharge of fecal blood ceased in 9 of 10 patients (*p* < 0.05). Butyrate enemas were tolerated without side effects.	W. Scheppach 1992 [88]
Nightly 60 mL SB enemas (80 mM) or isotonic saline placebo	38 active distal UC (left-sided ulcerative colitis) SB: 19, placebo: 19	SB was not efficacious. Clinical improvement (UCDAI drop ≥ 2 points) 37% (7/19) in SB, 47% (9/19) in placebo.	Clinical remission (UCDAI < 3) was achieved by 16% of patients in both the SB and placebo groups. No toxicity.	Steinhart 1996 [89]
Intrarectal enemas (100 mL SB, 80 mM s) plus 5-ASA (2 g). 4 weeks, twice daily Oral therapy (at stable dosages for at least two months) was maintained throughout the study	9 refractory distal UC unresponsive to standard therapy	Marked clinical and endoscopical improvement (7/9). Endoscopic scores reduced significantly (from 2.7 to 1.4). Histological scores also reduced (from 2.6 to 1.9).	Clinical improvement occurred relatively quickly, usually within the second week of therapy. Reduced bleeding and reduced diarrhea. The number of bowel movements per day decreased (from 6.2 to 2.1). Overall clinical results were rated as excellent or marked (7/9). One patient experienced a mild burning sensation in the rectum.	P. Vernia 1995 [90]
Topical enemas, Group A: 5-ASA (2 g) plus 80 mM SB, Group B: 5-ASA plus saline placebo, 6-week twice daily	51 distal UC 5-ASA +SB: 24 and 5-ASA +placebo: 27	Group A treatment was significantly more effective than B. Group A achieved remission in 6 patients vs. only 1 in Group B (*p* < 0.05). Significant superiority was noted for bowel movements (*p* < 0.01), urgency (*p* < 0.05), and patient self-evaluation (*p* < 0.01).	No adverse side effects. The data confirmed the utility of topical SB in achieving remission/marked improvement in refractory distal UC. SB is suggested as one of the active drugs needed to optimize therapeutic strategies.	Vernia 2003 [91]
Enemas of 100 mM SB enemas (n = 6) or placebo (n = 5), twice daily o 8 weeks. Existing oral medication was maintained	11 active distal UC	Significant inhibition of NF-kB (p65) in lamina propria macrophages (reduced from 77.7% to 11% after 8 weeks of butyrate). Significant reduction in the number of neutrophils (in crypt and surface epithelia) and lymphocytes/plasma cells in the lamina propria.	Significant decrease in DAI compared to entry (after 4 and 8 weeks). The DAI reduction after 8 weeks was significantly superior compared to the placebo group. Clinical and histological improvements were strongly associated with the inhibition of NF-kB activation.	Luhrs 2002 [92]
Daily rectal enema (60 mL) of 100 mmol/L SB or placebo (NaCl)	6 healthy volunteers. 35 UC	Expression of MUC2 (mucin) and TFF3 (trefoil factor 3), percentage of sialomucins, total mucin secretion, and secretory IgA (sIgA) concentrations were measured.	SB did not significantly modulate the expression of MUC2 (e.g., fold change 1.04/1.05) or TFF3 (e.g., fold change 0.91/0.94). The intervention did not affect sialomucins, mucin secretion, or sIgA concentrations.	Hamer 2010 [93]
Daily 60 mL rectal enemas containing 100 SB (n = 17) or saline (placebo, n = 18) administered over 20 days	35 UC	Minor effects on colonic inflammation and no significant overall effects on oxidative stress parameters. SB led to a significant increase in the colonic IL-10/IL-12 ratio and increased colonic concentrations of CCL5.	Clinical Activity Index (CAI), Endoscopic Grading System (EGS), histology score, FC, CRP, daily symptom score, feces consistency, and frequency did not significantly differ within or between SB and placebo groups.	Hamer 2010 [94]
SB enema 600 mmol/L twice daily for 30 days (10 patients in the treatment group)	20 colorectal cancer or diverticulitis	Significant improvement in endoscopic scores (*p* < 0.01); reduced/unchanged mucosal atrophy; upregulation of mucosal repair-related genes and BMP antagonists.	Prevention of deviated colon/rectum atrophy and improved tissue integrity recovery.	Luceri 2016 [95]

Microencapsulated sodium butyrate (BLM), capsules (Cps); UC disease activity index (UCDAI) 5-aminosalicylic acid (5-ASA), Crohn’s disease (CD), C-reactive protein (CRP), disease activity index (DAI), inflammatory bowel disease (IBD), Interleukin-10 (IL10), Interleukin-12 (IL12), Interleukin-1 beta (IL-1b), mucin glycoproteins (MUC2), Nuclear Factor kappa-light-chain-enhancer of activated B cells (NF-kB), sodium butyrate (SB), short-chain fatty acid (SCFA), ulcerative colitis (UC).

## Data Availability

No new data were created or analyzed in this study. Data sharing is not applicable to this article.
